# Effect of a Natural Eye Drop, Made of *Plantago Ovata* Mucilage on Improvement of Dry Eye Symptoms: A Randomized, Double-blind Clinical Trial

**DOI:** 10.22037/ijpr.2019.1100717

**Published:** 2019

**Authors:** Neda Haji-Ali-Nili, Fariba Khoshzaban, Mehrdad Karimi, Roja Rahimi, Elham Ashrafi, Reza Ghaffari, Ali Ghobadi, Mahmoud Jabarvand Behrouz

**Affiliations:** a *Traditional Medicine Clinical Trial Research Center, Shahed University, Tehran, Iran. *; b *Department of Iranian Traditional Medicine, School of Traditional Medicine, Tehran University of Medical Sciences, Tehran, Iran.*; c *Department of Traditional Pharmacy, School of Traditional Medicine, Tehran University of Medical Sciences, Tehran, Iran. *; d *Eye Research Center, Farabi Eye Hospital, Tehran University of Medical Sciences, Tehran, Iran.*; e *Department of Traditional Pharmacy, School of Traditional Medicine, Iran University of Medical Sciences, Tehran, Iran.*

**Keywords:** Dry eye disease, Persian medicine, *Plantago ovata*, Mucilage, Randomized controlled trial

## Abstract

Dry eye disease is a relatively common eye disorder associated with decrease in quality of life. In this study, efficacy of an eye drop of *Plantago ovata *mucilage on symptoms of dry eye disease was evaluated. In a randomized, double-blind, placebo-controlled clinical trial, sixty dry eye patients with ocular symptoms and total Ocular Surface Disease Index (OSDI) score of ≥12 were randomly assigned to receive either a natural ophthalmic drop, made of *Plantago ovata *mucilage or placebo 4 times a day for 6 weeks. The patients were evaluated at pretreatment (baseline), weeks 4 and 6 post-treatment. The evaluation of the efficacy and safety were conducted based on the OSDI questionnaire, the noninvasive tear film break-up time (NI-BUT) with keratograph, the Schirmer test without anesthesia, and the osmolarity test, as well as by monitoring possible adverse events. After 6 weeks, within group analysis showed a significant improvement in total OSDI score (*p *< 0.001). In addition, between group comparison revealed a significant improvement in the OSDI score of the intervention group (*p *< 0.001). Although, NI-BUT was significantly improved in the *Plantago ovata *group (*p *= 0.004), however no statistically significant difference was observed in between group analysis. There were no significant differences between two groups, or significant changes within the groups in the Schirmer test without anesthesia and the osmolarity test. No serious adverse events were reported. In conclusion, *P. ovata *mucilage is a natural, inexpensiveness, and safe lubricant polymer that could have beneficial ocular effects on subjective symptoms of the patients with dry eye disease.

## Introduction

Dry eye disease (DED) is a common complicated ocular disease that devastates tear film and ocular surface integrity (1). Bothersome ocular irritation symptoms such as dryness, burning, foreign body sensation, eye pain, and visual disturbances are common causes to seek eye care (2, 3). This may affect quality of life and work productivity of the patients (4-8). The estimated prevalence of DED is ranging from <0.1% to as high as 33% (9, 10). It has a multifactorial etiology with a challenging treatment. Two major etiopathogenic types of dry eye are aqueous-deﬁcient dry eye and evaporative dry eye (11). Regardless of DED subtypes, pathological events involved in ocular surface injury are similar. 

It is documented that, the underlying pathogenesis of DED comprises an inflammatory process mediated by T-cell lymphocytes (12). Two basic mechanisms of dry eye are tear hyperosmolarity and tear ﬁlm instability (11). Increased evaporation or decreased lacrimal ﬂow concludes to tear hyperosmolarity that stimulates the production of proinﬂammatory cytokines. Inﬂammatory mediators cause loss of goblet cells, decreased mucin production and subsequent instability of tear ﬁlm (13). This instability eventually reintensifies ocular surface hyperosmolarity (13-17). 

Lubricants, commonly prescribed as the ﬁrst-line management of dry eye, are suggested in all levels of disease severity (18). There are several formulations of lubricant eye drops that can adequately alleviate DED symptoms and provide treatment objectives. Given that mechanism of dry eye is an inﬂammatory process, formulation of artificial tears containing anti-inflammatory agents may effectively lead to improvement in the signs and symptoms of DED. The active ingredients of several newly developed chemical drugs, are natural components of plants that have useful properties including anti-oxidation, anti-inflammation, anti-apoptosis, and recovery of the body’s homeostasis (19-21).

The beneficial effects of herbal compounds on some ocular disease such as age-related macular degeneration (AMD), glaucoma, cataract, retinal disorders including diabetic retinopathy and retinitis pigmentosa as well as their protective effects against toxic agents, affecting human corneal epithelial cells, have recently been examined (20, 22-31). However, few clinical trials have been performed to study the effect of herbal medicines on DED (32-34). In Persian medicine (PM), several herbal products have previously been used for DED (35-37). Based on referral PM texts, particularly “the Canon of Medicine” (by Avicenna 980-1038 AD), *Plantago ovata *(*P. ovata*) was suggested as a natural remedy for treatment of dry eye (38). The* P. ovata* is known as a potent hydrating agent in PM and has been used for many different purposes like lubrication of lungs, polydipsia and fever. It has also been used for treatment of various diseases including constipation, gastrointestinal ulcers, hoarseness and inflammations (39, 40). Recent studies have confirmed many pharmacological effects for *P. ovate*. These include wound healing, antiulcer, bronchospasm, anti-inflammatory, and antioxidant effects (41-47). 

The objective of this study was to evaluate the effects of a herbal ophthalmic drop of *P. ovata* mucilage on improvement of dry eye disease in patients aged 30 to 60 years who were suffering from ocular symptom.

## Experimental

*Study design*

A randomized, double-blind, placebo–controlled study was conducted on 60 consecutive patients with dry eye disease in Farabi Eye Hospital (Tehran University of Medical Sciences, Tehran, Iran). Before any study –specific investigation, each participant provided a written informed consent form.

The Ethics Committee of Shahed University approved the protocol (approval number: 4/1196) before the study was initiated. Also, the trial was registered in the Iranian Registry of Clinical Trials with the registration number of IRCT 2015032621540N1.

*Study population*

Eligible patients were age 30-60 years with a history of dry eye disease for at least 6 months. In addition, they were required to meet the following inclusion criteria presenting at least one symptom of dryness, grittiness or foreign body sensation and Ocular Surface Disease Index (OSDI) questionnaire score ≥12. In addition, patients with a history of other eye diseases or ocular interventions (including pterygium, chemical eye burning, refractive surgery, punctual plug and punctual occlusion), chronic systemic disease (including thyroid eye disease, diabetes, and graft –versus- host disease), active eye infection, allergy to any ingredients of the study medicine, and those receiving any chronic systemic medications (antihistamines, antidepressants, diuretics and corticosteroids) or dose alterations during the study were excluded from the study. 

*Study intervention*

Patients were randomly allocated to receive either herbal eye drop or placebo (distilled water) four times a day.

Herbal eye drop was administered in 20 mL white drop bottles (containing 15 mL of solution) with white caps. Placebo was also supplied in identical drop bottles.

The patients were instructed to use their study medication (or placebo) in both eyes, four times a day. They were also allowed to use a preservative-free lubricant eye drop, four times daily, during the study period. The preservative-free lubricant eye drops (Artelac, Dr. Gerhard Mann, Chem.-pharm. Fabrik GmbH, Berlin, Germany) were supplied in unit dose containers. To avoid washing out the study medication, the patients were instructed to apply 10 min interval between instillation of the medication.

**Figure 1:**
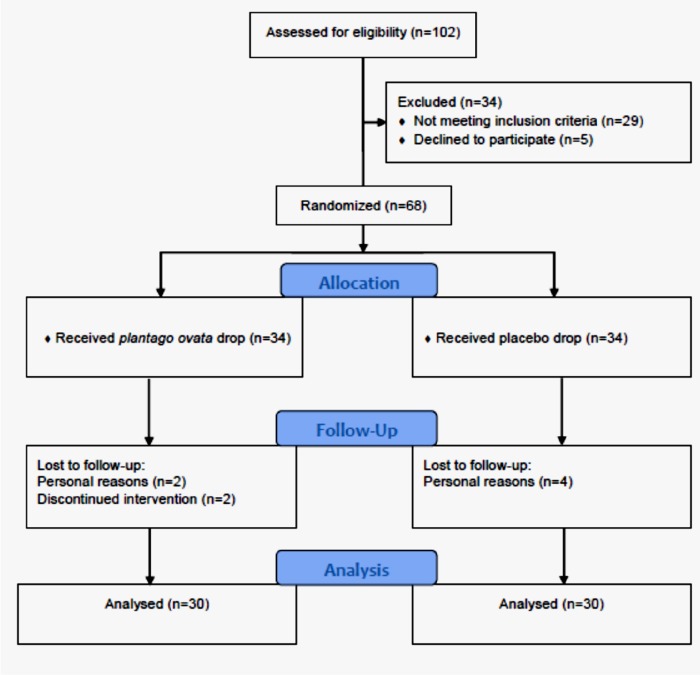
CONSORT flow chart of the study

**Table 1:** Patient demographics and baseline disease characteristics

**Variable**	***P. ovata***	**Placebo**	***P***
Age (years) (Mean ± SD)	48.5 ± 9.8	46.07 ± 12.04	0.39
Female/Male, n	24/6	23/7	0.75
**Educational level**			
≤ High school, n	26	23	0.67
≥ Bachelor’s degree	4	7	
OSDI score baseline (Mean ± SD)	47.5 ± 17.85	43.06 ± 15.89	0.31
Breakup time score baseline, s (Mean ± SD)	9.59 ± 5.56	9.77 ± 6.78	0.09
Schirmer score baseline, s (Mean ± SD)	9.91 ± 9.05	13.07 ± 9.7	0.74
Osmolarity score baseline (Mean ± SD)	300.52 ± 5.56	305.61 ± 18.77	0.71

n: Number; s: Second.

**Table 2:** The subjective treatment effects

**Test**			***P. ovata***	**Placebo**	***P *** **(between groups)**
	Baseline (Mean ± SD)		47.5 ± 17.85	43.06 ± 15.89	<0.0001
OSDI total	6 week after treatment (Mean ± SD)		18.39 ± 8.95	36.5 ± 16.58	
*P *(within group)			<0.0001	<0.0001	
Sensitive to light		Baseline (Mean ± SD) 6 week after treatment(Mean ± SD)	3.33 ± 0.84 1.33 ± 0.95	2.46 ± 1.40 0.000	1.96 ± 1.29
*P *(within group)			<0.0001	0.005	
Grittiness		Baseline (Mean ± SD) 6 week after treatment (Mean ± SD)	2.63 ± 1.32 0.80 ± 0.80	2.63 ± 1.35 0.000	2.06 ± 1.28
*P *(within group)			<0.0001	<0.0001	
Burning		Baseline (Mean ± SD) 6 week after treatment (Mean ± SD)	1.80 ± 1.42 0.43 ± 0.62	1.83 ± 1.17 0.000	1.33 ± 0.99
*P *(within group)			<0.0001	<0.0001	
Baseline (Mean ± SD)			1.03 ± 1.09	0.99 ± 1.02	

P. ovata: plantago ovate.

**Table 3:** The objective treatment effects

**Test**		***P. ovata***	**Placebo**	***P *** **(between groups)**	
	Baseline (Mean ± SD)	9.59 ± 5.56	9.77 ± 6.78		
NI-BUT 6 week after treatment (Mean ± SD)		12.75 ± 6.25	9.42 ±	6.52 0.09	
*P *(within group)		0.004	0.856		
Baseline (Mean ± SD) Schirmer test 6 week after treatment (Mean ± SD)		9.91 ± 9.05 12.1 ± 10.81	13.07 ± 9.73 11.65 ± 9.03		0.74
*P *(within group)		0.15	0.46		
Baseline (Mean ± SD)		300.52 ± 5.56	305.61 ± 18.77		

*P. ovata: plantago ovate*; NI-BUT: Noninvasive tear ﬁlm break-up time.

*Formulation and standardization of herbal eye drop*

Seeds of the *Plantago ovata* (family *Plantaginaceae*) were identiﬁed by an expert staff botanist, and are kept at the herbarium of the School of Pharmacy, Tehran University of Medical Sciences, under the voucher number PMP-751. Eight grams of *Plantago ovata* husk was soaked in 100 mL distilled water for 12 h. Then, the material was squeezed through muslin cloth for filtering and extracting out the mucilage content. 

The separated mucilage was dried in oven at temperature less than 60 °C, and then was powdered and stored in a desiccator. For preparation of the eye drop solution, 1 g of mucilage powder was dissolved in 100 mL distilled water and then filled in 20 mL drop containers. Autoclaving at 121 °C for 15 min was used for sterilization of the final product (49). For standardization of herbal drop, total polysaccharide content of the solution was determined according to the procedure of Dubois *et al.* (50). The total polysaccharide content of *P. ovata* eye drop was found to be 0.35 µg/mL (mean of three determinations).

*Randomization*

Eligible patients were randomly assigned corresponding to allocation code for drug and placebo groups by using a block –randomization list (non–stratified with equal–length blocks) obtained by Microsoft Excel 2010 program. 

*Study outcomes*

Selected patients enrolled in the study and underwent one baseline and 2 follow up evaluations. At follow up1 (baseline), patients signed an informed consent and their symptoms were assessed according to OSDI questionnaire. The OSDI questionnaire is a 12-item questionnaire with score ranging from 0 to 100 and contains three subscales: ocular discomfort symptoms (OSDI- symptoms), vision-related function (OSDI- function) and environmental triggers (OSDI –triggers) (4). At this stage, the objective symptoms were also examined with this order: Osmolarity test with Tear Lab Osmolarity System (TearLab Corp), Noninvasive tear ﬁlm break-up time (NI-BUT) with keratograph, measured by OCULUS Keratograph 5M), and Schirmer test without anesthesia. The same procedure and outcome determination were carried out at the follow up3, in the week 6 of intervention (end of study). 

At follow up2, which was a call visit at week 4 of the treatment period, the patients were asked to report any symptoms after the initiation of treatment, as well as duration and the severity of the symptoms. The severity of symptoms were evaluated as mild (no action taken), moderate (limiting daily activities such as reading, driving at night, watching TV and working on the computer), or severe (limiting personal activities). 

Safety evaluation included caring about any possible adverse events, and examining visual acuity and ophthalmologic examinations using slit-lamp.

*Sample size estimation and Statistical analyses*

A sample size of 34 in each group was estimated with α = 0.05 and the study power of 80%. Data normality was assessed using the Shapiro-Wilk test. The Mann-Whitney *U* test was used to compare differences between groups, when their distributions were not normal. 

Similarly, within-group treatment effects (before and after intervention) were compared using the Wilcoxon test. *P*-values < 0.05 were considered as statistically significant. All of the statistical analyses were performed using statistical package for the social sciences, version 19.0 (SPSS Inc., Chicago, IL, USA).

## Results

From May 24, 2015 to September 18, 2016, in total 102 patients, referred to the ocular surface clinic at the Farabi Eye Hospital, were eligible to be recruited for this study. Overall, 60 patients, 30 cases in the *P. ovata* group and 30 cases in the placebo group completed the trial (Figure 1). Demographics and baseline characteristics of the study groups are presented at the Table 1.

*Efficacy evaluation*

*Subjective efficacy evaluation*

Within groups analyses revealed that in both groups, there are significant reduction in the OSDI (total score) after 6 weeks of the study period (*P *< 0.0001). Also, between groups comparisons showed that improvement of the OSDI (total score) in the *P. ovata* group is greater than that of the placebo group after 6 weeks of drug use (*P* = 0.0001) (Table 2).

Similar significant improvements (*P* < 0.006) were obtained for the 4 symptoms belonging to OSDI- symptoms subscales of the OSDI questionnaire including sensitivity to light, grittiness of the eyes, soreness of the eyes and blurred vision with an exception for the within group changes of the blurred vision in the placebo group (*P* = 0.49) (Table 2). Poor vision, as the fifth symptom of OSDI- symptoms subscales in OSDI questionnaire did not have any significant changes (Table 2).

*Objective efficacy evaluation*

Regarding 3 objective treatment effects comprising the NI-BUT, the Schirmer test and the tear film osmolarity test, results were not encouraging. Although, in the intervention group an improvement in the NI-BUT was seen after 6 weeks of using ophthalmic drop of *P. ovata* mucilage (*P *= 0.004), however, this was not confirmed by between groups comparisons (Table 3).

*Safety evaluation*

There were no serious adverse events in either study groups. The most common adverse reactions reported were burning in the *P. ovata *(n = 5, (17%)) and the placebo groups (n = 3, (10%)). Others were blurred vision, occurring in ten minutes after drug use, with the frequency of n = 3 (10%) and n = 1, (0.3%) in the *P. ovata *and placebo groups, respectively. 1 patient (0.3%) in the *P. ovata *group had conjunctival hyperemia.

The burning and blurred vision symptoms were tolerable, causing no discontinuation in medication use. With regard to local ocular tolerance parameters assessed during ophthalmologic examination, no major differences between two groups were found.

## Discussion

This study evaluated the effects of an ophthalmic drop made of* P. ovata* mucilage in patients with dry eye disease, using a double-blind randomized clinical trial. 

Our findings showed signiﬁcant improvements in the subjective symptom of dry eye disease in patients using ophthalmic drop of* P. ovata* mucilage. However, our findings were not in favor of the herbal drop, supporting its beneficial impact on the objective symptoms of dry eye disease. 

Clinical and epidemiological studies have showed that dry eye disease is more prevalent in females than males (51, 52). Likewise, majority (78%) of the patients entered in this study were females. 

Our study herbal product could signiﬁcantly improve the total OSDI score and the symptoms of sensitivity to light, grittiness, sore eyes, and blurred vision. This may possibly be related to the potentially hydrating, anti-inflammatory, and antioxidant effects of *P. ovata*.

The first stage in the treatment of dry eye disease accompanied by reduction in lacrimal tear secretion and tear deficiency is hydrating and increasing tear volume. *P. ovata* husk has a high capacity of water absorption and thus could act as a potent hydrating agent (53).

Moreover, one of the applications of *P. ovata* mucilage in pharmaceutical industry is serving as water retention agent (54). Therefore, it can play as tear trapping agent in eye and inhibiting tear from evaporation.

Potential mechanisms by which *P. ovata* mucilage may improve ocular surface disease in dry eye were not evaluated in this study, but anti-inflammatory effects of *P. ovata *mucilage have been demonstrated in several studies. The two main mechanisms involved in dry eye disease are increase in tear osmolarity and ocular surface inflammation. Hyperosmolarity stimulates a cascade of inflammatory events in the epithelial surface cells involving mitogen-activated protein kinases (MAPKs) and nuclear factor kappa B (NF-*κB*) signaling pathways, as well as the generation of inflammatory cytokines such as caspase-1, interleukin (IL)-1*α*, IL-1*β*, and tumor necrosis factor-alpha(TNF-*α*). Activation of inflammatory mediators leads to cellular apoptosis, glycocalix mucin loss, and epithelial damage that result in tear film instability. The severity of disease is also affected by inflammation (1, 55). Decrease in the level of caspase 3 has been reported by *P. ovata* (44). IL-8 and NF-κB are other inflammatory mediators that their concentrations could be affected by* P. ovata *(47). It has also been demonstrated that decrease in some pro-inflammatory mediators including nitric oxide, leukotriene B4, and TNF-*α* after dietary supplementation with *P. ovata *can occur (45). Other anti-inflammatory activities reported from *P. ovata* include inhibition of the protein kinase C, down-regulation of the expression of intercellular adhesion molecule-1 (ICAM-1), as well as inhibition of the inflammation produced by 5-hydroxy-6,8,11,14-eicosatetraenoic acid and leukotriene B4 (46).

In addition, the antioxidant activity is another property of *P. ovata* seed. Oxidative stress can be a causative factor in the development of dry eye disease as well as inflammation. It is documented that mitochondria-induced oxidative damage in the lacrimal glands induces lacrimal dysfunction, resulting in dry eye disease (56). Nakamura *et al.* have suggested a relationship between the accumulation of oxidative stress and the etiology of corneal epithelial alterations in blink-suppressed dry eyes (57). A significant reduction in myeloperoxidase activity and restoration of glutathione levels have been shown after supplementation with *P. ovata* seed (45, 48).

As the inflammation and oxidation process are the bases for pathophysiology of the dry eye disease, significant improvement of ocular symptoms of dry eye disease by ophthalmic drop of *P. ovata* mucilage, which was confirmed by the OSDI questionnaire, is reasonable. 

On the other hand, we did not find a significant priority for the study herbal drop in improvement of the objective symptoms of dry eye in comparison to the placebo. This could be due to the small sample size of the study, technical complications to perform the study tests, and the short duration of the follow up period. There are studies reporting similar controversial findings for the subjective and objective symptoms in patients with dry eye disease (58-60).

In conclusion*, P. ovata* mucilage is a natural, inexpensiveness, and safe lubricant polymer that could have beneficial ocular effects on symptoms of the patients with dry eye disease. 
